# Methodology Assessment of Endoscopic Ultrasound Radiofrequency Ablation (EUS‐RFA) for Pancreatic Neoplasms: Results From an International Survey

**DOI:** 10.1111/den.70212

**Published:** 2026-06-29

**Authors:** Andrea Lisotti, Matteo Tacelli, Stefano Francesco Crinò, Graziella Masciangelo, Pietro Fusaroli, Marc Barthet, Nirav Thosani, Maria Cristina Conti Bellocchi, Sarah Leblanc, Tawfik Khoury, Paolo Giorgio Arcidiacono, Antonio Facciorusso, Fabrice Caillol, Marc Giovannini, Lorenzo Fuccio, Jayanta Samanta, Anthony Y. B. Teoh, Takeshi Ogura, Roald Flesland Havre, Somashekar G. Krishna, Bojan Kovacevic, Vinay Dhir, Peter Vilmann, Sundeep Lakhtakia, Bertrand Napoléon, Khanh Do‐Cong Pham, Abed H. Al‐Lehibi, Mohammad A. Al‐Haddad, Nawaf O. Alotaibi, Michele Amata, Andrea Anderloni, Elia Armellini, Cecilia Binda, Federica Borrelli de Andreis, Ivan V. Borbath, Angelo Bruni, Silvia Carrara, Lucio Carrozza, Antoine Charachon, Ulriikka Chaput, Germana De Nucci, Pierre H. Deprez, Francesco Maria Di Matteo, Mohamad Ali Eloubeidi, Carlo Fabbri, Mohamed Gasmi, Paraskevas P. Gkolfakis, Sebastien Godat, Joan B. Gornals, Frank G. Gress, Philippe Grandval, Gregory B. Haber, Zaher S. Houmani, Julien Jézéquel, Mairin Joseph‐Talreja, Petko Ivanov Karagyozov, Evangelos Kalaitzakis, Stéphane Koch, Alberto Larghi, Marc Le Rhun, Kevin Marcel, Aurelio Mauro, Leonardo Minelli Grazioli, Mehdi Mohamadnejad, Laurent Monino, Pedro G. Moutinho‐Ribeiro, Laurent Palazzo, Nonthalee Pausawasdi, Katarzyna M. Pawlak, Stephen P. Pereira, Marcin Polkowski, Frederic P. Prat, Jean‐Philippe Ratone, Paul Rivallin, Gianenrico Rizzatti, Gemma Rossi, Marco Sacco, Ali A. Siddiqui, Ajaypal Singh, Leonardo Sosa‐Valencia, Serena Stigliano, Daniela Tabacelia, Ilaria Tarantino, Geoffroy Vanbiervliet, Rogier P. Voermans, Thomas R. de Wijkerslooth, Anna Wiechowska‐Kozlowska, Piera Zaccari

**Affiliations:** ^1^ Gastroenterology and Digestive Endoscopy Unit, Imola Hospital, AUSL Imola, and Department of Medical and Surgical Sciences (DIMEC) University of Bologna Bologna Italy; ^2^ Department of Gastroenterology and Digestive Endoscopy Hôpital Privé Jean Mermoz, Ramsay Santé Lyon France; ^3^ Pancreatobiliary Endoscopy and Endosonography Division IRCCS San Raffaele Hospital, Vita‐Salute San Raffaele University Milan Italy; ^4^ Department of Medicine University of Verona Verona Italy; ^5^ Department of Gastroenterology and Digestive Endoscopy Hôpital Nord, Assistance Publique–Hôpitaux de Marseille, Aix‐Marseille University Marseille France; ^6^ Division of Gastroenterology, Hepatology and Nutrition McGovern Medical School at UTHealth Houston Texas USA; ^7^ Department of Gastroenterology Galilee Medical Center Nahariya Israel; ^8^ Faculty of Medicine in the Galilee Bar‐Ilan University Safed Israel; ^9^ Gastroenterology Unit, Department of Experimental Medicine University of Salento Lecce Italy; ^10^ Department of Gastroenterology and Digestive Endoscopy Institut Paoli‐Calmettes, Aix‐Marseille University Marseille France; ^11^ Department of Endoscopy and Endoscopic Ultrasound Institut de Recherche contre les Cancers de l'Appareil Digestif (IRCAD) Strasbourg France; ^12^ Gastroenterology and Digestive Endoscopy Unit IRCCS Azienda Ospedaliero‐Universitaria di Bologna; University of Bologna Bologna Italy; ^13^ Department of Gastroenterology Postgraduate Institute of Medical Education and Research (PGIMER) Chandigarh India; ^14^ Department of Surgery Chinese University of Hong Kong Hong Kong SAR China; ^15^ Endoscopy Center Hong Kong Sanatorium and Hospital Hong Kong SAR China; ^16^ Second Department of Internal Medicine Osaka Medical and Pharmaceutical University Osaka Japan; ^17^ Department of Gastroenterology Haukeland University Hospital Bergen Norway; ^18^ Department of Clinical Medicine University of Bergen Bergen Norway; ^19^ Division of Gastroenterology, Hepatology and Nutrition The Ohio State University Wexner Medical Center Columbus Ohio USA; ^20^ Department of Gastrointestinal and Hepatic Diseases Copenhagen University Hospital – Herlev and Gentofte Herlev Denmark; ^21^ Department of Gastroenterology and Digestive Endoscopy SL Raheja Hospital Mumbai India; ^22^ Department of Gastroenterology Asian Institute of Gastroenterology Hyderabad India

## Abstract

**Background and Aims:**

Endoscopic ultrasound‐guided radiofrequency ablation (EUS‐RFA) is increasingly used; however, clinical application remains unstandardized. We assessed real‐world practice among international users.

**Methods:**

A cross‐sectional 70‐item survey was conducted. Results are presented descriptively (numbers, percentages).

**Results:**

Overall, 91 of 175 invited physicians from Europe (74.4%), North America (13.3%), and Asia (12.2%) completed the survey. EUS‐RFA was performed by 94.1% of respondents for insulinoma, with heterogeneous responses for other indications. Most physicians (96.3%) performed EUS‐RFA under deep sedation or general anesthesia; marked variability was reported on antibiotic prophylaxis (57.5%), aggressive hydration (52.5%), generator power settings, ablation strategy, or probe selection. Lesions involving or located ≤ 1 mm from the main pancreatic duct were considered high risk by 97.5% and 85.0%, respectively, yet no standardized preventive strategy was identified. Post‐procedural management and follow‐up were heterogeneous, with a high proportion of responses for definitions of technical success (88.8%), clinical success in insulinoma (92.3%), and disease recurrence (97.5%), with high variability for definitions of partial ablation and post‐RFA pancreatitis.

**Conclusions:**

Despite the global expansion of EUS‐RFA, clinical practice remains highly heterogeneous and geographically skewed. The lack of standardized methodology and terminology poses significant barriers to generating high‐quality evidence.

## Graphical abstract

An international survey on endoscopic ultrasound‐guided radiofrequency ablation (EUS‐RFA) for pancreatic neoplasms reveals substantial heterogeneity in clinical indications, procedural techniques, peri‐procedural management, follow‐up strategies, and terminology among expert users, underscoring the critical need for international methodological standardization

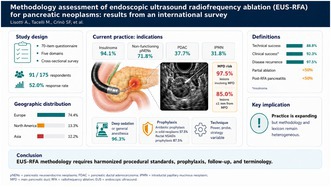

## 1 Introduction

Endoscopic ultrasound radiofrequency ablation (EUS‐RFA) is a minimally invasive ablative technique that uses high‐frequency alternating current to generate high temperatures; the EUS‐RFA energy is delivered through a dedicated electrode needle to induce coagulative necrosis (direct effect) and T‐cell‐mediated response (indirect effect) within target neoplasm [1].

Clinical applications and EUS‐RFA use are increasing, mainly for pancreatic ablation. To date, robust evidence is available on technical feasibility and safety profile. In contrast, clinical and oncological outcomes are supported by low‐level evidence [2, 3]. Thus, no guideline recommends EUS‐RFA as first‐line treatment for pancreatic neoplasms, even though recommendations differ substantially by indication: ENETS allows consideration of EUS‐RFA for selected localized insulinomas in high‐risk patients. For pancreatic ductal adenocarcinoma (PDAC), major international guidelines (ESMO and NCCN) emphasize systemic therapy and surgical resection when feasible, while locoregional ablative techniques are not recommended in routine clinical practice and remain investigational. On the other hand, AIOM does not recommend locoregional ablation for PDAC outside clinical trials. European, Kyoto, and ACG cyst guidelines restrict pancreatic cystic neoplasms ablation to trial settings. Finally, major renal cell carcinoma guidelines discuss local therapy or metastasectomy for selected metastatic disease, but do not provide pancreas‐specific recommendations for EUS‐RFA of pancreatic metastases [4, 5, 6, 7, 8, 9, 10, 11, 12].

During EUS‐RFA procedure, a dedicated needle is advanced into target lesion, avoiding contact of the active tip with interposed vessels, ducts, or gastrointestinal wall. Energy is delivered at different power settings, usually 10–50 W, and maintained for a few seconds before stopping once impedance thresholds are reached. Multiple ablations during the same session or multiple sessions can be performed to achieve complete ablation [13, 14, 15].

Despite the increasing EUS‐RFA use, a lack of standardization persists. Key aspects, such as indications and patient selection, prophylactic measures, generator settings, procedural setup and techniques, post‐treatment follow‐up, and even lexicon vary widely across centers [16, 17, 18, 19, 20, 21, 22]. Variations in local expertise and institutional protocols partially account for this heterogeneity, which could impair data interpretation and comparison across studies [23, 24, 25, 26, 27, 28].

This study aims to assess the level of standardization in EUS‐RFA across indications, procedural management, clinical and oncological follow‐up, and lexicon among international experts.

## 2 Methods

### 2.1 Study Design

Single‐round cross‐sectional survey without interactions and controlled feedback was designed. A questionnaire was created and sent via email to endosonographers identified through a comprehensive register of world users provided by the probe manufacturer (Taewoong Medical, Seoul, South Korea) in December 2024. Moreover, the corresponding authors of available EUS‐RFA bibliographic references have been invited; finally, the participants were asked to suggest any further interested physicians to be invited. The invitation email and questionnaire description are available in Appendices S1 and S2.

### 2.2 Statistical Analysis and Definitions

Qualitative results were expressed as numbers and percentages. Subgroup analysis was conducted by physician's region of origin. A post hoc sensitivity analysis has been conducted to determine if consensus levels differ according to operators' experience: answers from highly experienced users (> 20 EUS‐RFA cases) have been assessed to identify possible experience‐dependent practice patterns. The cut‐off > 20 procedures has been pragmatically chosen and should be interpreted cautiously, as no validated learning curve thresholds for EUS‐RFA have been evaluated. Subgroup comparisons according to geographic region and operator experience were performed on an exploratory basis using chi‐square testing. For indication‐level analyses across geographic regions, Bonferroni correction was applied to account for multiple comparisons. Both unadjusted and Bonferroni‐adjusted *p* values are reported; however, statistical significance was interpreted on the basis of Bonferroni‐adjusted *p* values, with adjusted *p* < 0.05 considered statistically significant.

## 3 Results

Invitation for participation was sent in January 2025 to 125 physicians, followed by another invitation to 50 physicians identified as described above. After 4 and 12 weeks, a reminder was sent; finally, 91 of 175 invited physicians (52.0%) completed the survey by August 2025.

### 3.1 Domain 1—Physician and Center Characteristics

Most participants (74.4%) worked in Europe, 13.3% from North America, and 12.2% from Asia. Forty‐one (45.1%) physicians have performed > 20 EUS‐RFA, 27 (29.6%) 10–20 EUS‐RFA, and the remaining 31 (34.0%) reported fewer than 10 procedures. A detailed description of the centers' volume and operators' characteristics is provided in Table 1. Table 2 summarizes local settings and units availability.

**TABLE 1:** Description of center and operators' experience (questionnaire domain 1).

Center volume		Personal experience	
*Professional setting*		*Board certification*	
Academic institution	63.3%	Gastroenterology	95.6%
Public hospital	20.0%	Surgery	4.4%
Private practice	13.3%	*EUS tissue sampling experience*	
*EUS volume*		< 5 year	9.9%
< 250 EUS/year	4.4%	5–10 years	25.3%
250–500 EUS/year	21.1%	> 10 years	64.8%
501–1000 EUS/year	37.8%	*EUS tissue sampling volume*	
> 1000 EUS/year	36.7%	< 50 EUS sampling/year	3.3%
*EUS‐RFA operators*		50–100 EUS sampling/year	13.3%
1 operator	36.7%	> 100 EUS sampling/year	83.3%
2 operators	38.9%	*EUS‐RFA experience (overall)*	
> 2 operators	24.4%	< 10 EUS‐RFA procedures	34.1%
*EUS‐RFA/year*		10–20 EUS‐RFA procedures	20.9%
< 5 EUS‐RFA/year	32.2%	20–50 EUS‐RFA procedures	23.1%
5–10 EUS‐RFA/year	24.4%	> 50 EUS‐RFA procedures	22.0%
11–15 EUS‐RFA/year	16.7%	*EUS‐RFA volume*	
> 15 EUS‐RFA/year	26.7%	< 5 EUS‐RFA/year	35.2%
*Center of Excellence for NEN Accreditation*	35.6%	5–10 EUS‐RFA/year	35.2%
		> 10 EUS‐RFA/year	29.7%

Abbreviations: EUS, endoscopic ultrasound; EUS‐RFA, EUS‐guided radiofrequency ablation; NEN, neuroendocrine neoplasm.

**TABLE 2:** Description of available facility at each center.

Units available at the same center	%
Gastrointestinal endoscopy unit	98.9
Gastroenterology ward	85.6
Endoscopic Retrograde Cholangiopancreatography (ERCP)	96.7
24/7 on call gastrointestinal endoscopy	91.1
24/7 radiology (included computed tomography scan)	92.2
Interventional radiology	86.7
General surgery	94.4
24/7 urgent surgery	86.7
Pancreatic surgery	78.9
Oncology unit	88.9
Pathology department	87.8
Endocrinology unit	80.0
Nuclear medicine	81.1
Multidisciplinary team (dedicated to gastrointestinal neoplasms)	92.2
Multidisciplinary team (dedicated to pancreatic neoplasms)	78.9
Multidisciplinary team (dedicated to neuroendocrine neoplasms)	56.7

### 3.2 Domain 2—EUS‐RFA Indications

The most common indications are pNENs, followed by pancreatic metastases, PDAC, and intraductal papillary mucinous neoplasm (IPMN) as reported in Table 3.

**TABLE 3:** Indication for endoscopic ultrasound‐guided radiofrequency ablation (EUS‐RFA).

Question 20: Indication for EUS‐RFA in your practice (multiple answers allowed)	All users (%)	Highly experienced users^a^ (%)
Insulinoma	94.1	94.7
Other functioning pNENs	54.1	60.5
Non‐functioning pNENs	71.8	84.2
Pancreatic metastases	57.7	63.2
Pancreatic ductal adenocarcinoma	37.7	52.6
IPMN	31.8	44.7
Liver tumors, including metastases	22.4	36.85
Abdominal lymph node metastases	12.9	23.7
Other solid pancreatic neoplasms	10.6	15.8
Other pancreatic cystic neoplasms	10.6	15.8
Adrenal gland tumor	10.6	15.8
Solid pseudopapillary tumor	9.4	7.9
Celiac plexus RFA	24.7	34.2

Abbreviations: EUS‐RFA, endoscopic ultrasound‐guided radiofrequency ablation; IPMN, intraductal papillary mucinous neoplasms; pNENs, pancreatic neuroendocrine neoplasms; RFA, radiofrequency ablations.

^a^ Highly experienced users have been defined as operators with more than 20 EUS‐RFA performed.

Regional differences in indications are reported in Table S1. After Bonferroni correction for multiple comparisons, statistically significant regional differences were observed only for PDAC and insulinoma indications. The use of EUS‐RFA for PDAC differed across regions, being reported by 26.6% of European, 83.3% of North American, and 55.6% of Asian respondents; this difference remained statistically significant after Bonferroni correction (adjusted *p* = 0.003). EUS‐RFA use for insulinoma also differed across regions, being reported by 96.9% of European, 75.0% of North American, and 100% of Asian respondents, and remained significant after correction (adjusted *p* = 0.038). A higher proportion of NF‐pNEN indications in North America, a lower proportion of pancreatic metastasis indications in Asia, and a higher proportion of IPMN indications in North America; however, these differences did not remain statistically significant after Bonferroni correction and should therefore be interpreted cautiously.

These findings suggest that clinical practice is influenced by regional factors, including local expertise, guideline interpretation, and healthcare systems. EUS celiac plexus RFA is performed by 41.7% respondents from North America, 18.8% from Europe, and 22.2% from Asia. When analyses were restricted to highly experienced users, no clear experience‐dependent indication pattern was identified.

Table 4 shows the indications for EUS‐RFA in patients suffering from functioning and NF‐pNENs, showing moderate clustering of responses (77.7%) for insulinoma in “selected cases after MDT discussion.”

**TABLE 4:** EUS‐RFA indications in patients with pancreatic neuroendocrine neoplasms.

Insulinoma		Non‐functioning pNENs	
Indication for EUS.RFA	%	Indication for EUS.RFA	%
Selected cases after MDT discussion	77.7	Selected cases after MDT discussion	71.8
High‐risk surgical patients	56.5	High‐risk surgical patients with indication for surgery	62.4
Based on patients' preference	38.8	Increasing tumor size (but still ≤ 20 mm)	45.9
Always	9.4	Based on patients' preference	40.0
Only within research protocols	8.2	Only within research protocols	5.9
Never	2.3	Never	16.5

Abbreviations: MDT, multidisciplinary team; pNEN, pancreatic neuroendocrine neoplasm.

Among physicians performing EUS‐RFA for NF‐pNENs, 50.0% of them used to treat only G1 tumors, 30.5% both G1 and G2 tumors, while the remaining 19.5% used to treat NF‐pNENs independently from grading. Regarding a minimum tumor size for EUS‐RFA in pNENs, 13.2% of physicians stated that there is no minimum pNEN size, 11.0% suggested a minimum size between 1 and 5 mm, 27.5% between 6 and 10 mm, and 25.3% suggested a minimum size ≥ 10 mm. On the other hand, we observed moderate clustering of responses (85.7%) in identifying 20–24 mm as the maximum size for pNENs, while 5.5% suggested a maximum size between 15 and 19 mm, 5.5% between 25 and 29 mm, and the remaining 3.3% between 30 and 40 mm. Marked variability was reported for indications in other neoplasms than pNENs (Table S2). In patients with pancreatic metastases, 71.4% of physicians use EUS‐RFA among “selected cases after MDT discussion,” while 50.6% and 57.7% of physicians did never consider EUS‐RFA in patients with PDAC and IPMN, respectively.

### 3.3 Domain 3—Procedural Management

High proportion of responders (96.3%) performed EUS‐RFA under deep sedation or general anesthesia; 77.6% performed EUS‐RFA in an inpatient setting (61.3% due to clinical considerations, while 16.3% due to reimbursement issues). When asked whether it is recommended to perform EUS‐RFA in an X‐ray‐equipped endoscopic room, 81.2% of physicians consider it unnecessary.

When asked about antibiotic prophylaxis, 57.5% responders reported a routine in solid neoplasms, while 76.3% in cystic neoplasms; similar percentages (62.2% and 75.6%, respectively) were found among highly experienced operators. 87.5% of physicians used rectal non‐steroidal anti‐inflammatory drugs (NSAIDs) and 52.5% aggressive hydration to prevent post‐RFA pancreatitis; similar proportions (86.5% for NSDAIDs, 46.0% for aggressive hydration) among highly experienced operators. Lastly, very‐low amount of users (46.3% and 37.8% among highly experienced operators) administrated a combination of both prophylactic NSAIDs and aggressive hydration.

Notably, a clinically relevant discrepancy emerged between risk perception and management of lesions involving or located close to the main pancreatic duct (MPD); no standardized preventive strategy was identified, with highly heterogeneous approaches regarding power modulation, prophylactic stenting, or avoidance of EUS‐RFA. Most physicians (97.5% and 97.3% among highly experienced users) were concerned about the risk of post‐RFA adverse events in case of neoplasms involving the MPD. Of them, 85.0% (78.4% among highly experienced users) were concerned about lesions ≤ 1 mm from the MPD, while 51.3% for lesions ≤ 2 mm from the MPD. A great variability was found on the management (power setting, prophylactic MPD stenting, avoiding EUS‐RFA) of neoplasms located close to the MPD, with or without involvement (Table S3).

75.0% of physicians used contrast‐enhanced EUS (CH‐EUS) after EUS‐RFA, and 72.5% before ablation. Of these, SonoVue (Bracco, Italy) was used by 88.6% of respondents, mainly reflecting local availability of ultrasound contrast agents. High variability was reported on the time that should elapse.

Among all responders, 59.0% use the same power setting for all EUS‐RFA procedures; a similar percentage of 56.8% was found among highly experienced users. Table 5 summarizes the relative use of different power settings for each EUS‐RFA indication; in detail, 66.6% used the 50‐W power setting for ablation of pNENs, while no clustering was observed in any other condition. No standardization on the preferred ablation strategy: 46.3% use multiple high‐power quick shots, 23.8% use single or a few low‐power, longer shots, and 30.0% have no predefined technical strategy.

**TABLE 5:** Endoscopic ultrasound generator power setting used for different indications.

Power setting	Same setting for all EUS‐RFA	pNENs (%)	Metastases (%)	PDAC (%)	IPMN (%)
< 30 W	None	7.7	24.2	16.5	19.8
30–49 W	33.3%	26.4	29.7	49.5	45.1
≥ 50 W	66.6%	49.5	46.2	34.1	35.2
Depending on tumor size and probe length	None	16.5	None	None	None

Abbreviations: EUS‐RFA, endoscopic ultrasound‐guided radiofrequency ablation; IPMN, intraductal papillary mucinous neoplasm; PDAC, pancreatic ductal adenocarcinoma; pNENs, pancreatic neuroendocrine neoplasms.

Most responders (72.5%) selected the probe length based on tumor size and location, with a small percentage that used the same tip length (10 mm) for all EUS‐RFA procedures. Although the 10‐mm tip probe is widely used (91.0%), 51.3% of physicians use the 5‐mm tip probe, and < 50% use the 7‐mm or 15‐mm tip probes. The same proportions have been found among highly experienced users.

71.3% of responders (67.5% among highly experienced users) stated that there is not a maximum number of applications per EUS‐RFA session, repeating energy delivering until all tissue seems ablated. 12.1% (6.2% among highly experienced users) stopped after 3 applications, while 11.0% (25.2% among highly experienced users) performed more than 5 applications per session.

Regarding EUS‐RFA application time, 57.0% of physicians (46.0% among highly experienced users) applied EUS‐RFA energy until the generator automatically stops or impedance rapidly increases, with no maximum time, while 35.4% of physicians (43.2% among highly experienced users) manually stop the generator when hyperechoic white bubbles appear within the lesion.

### 3.4 Domain 4—Post‐Procedural Management

Testing serum biochemistry on the same day as EUS‐RFA was not considered necessary by 82.3% of physicians; on the other hand, 53.2% routinely test serum biochemistry the day after the procedure. 95.6% suggested testing complete blood cell counts, 91.1% fasting glucose in patients with insulinoma, 82.2% amylase or lipase, and 57.8% for C‐reactive protein.

Routine CT scan with contrast medium before discharge or within 2 weeks was deemed unnecessary by 72.1% of physicians, whereas 27.9% performed a routine CT scan to rule out adverse events or assess the presence of residual vital tissue.

50.7% physicians recommended a 3‐month examination as the ideal timing for the first oncologic follow‐up in patients with pNENs; 33.8% of physicians suggested a 1‐month follow‐up, while 15.6% suggested a 6‐month follow‐up. No optimal timing for defining final oncologic treatment outcomes in patients with pNENs. 80.0% physicians suggested using CT with contrast medium, and 57.5% suggested EUS. Marked dispersion of answers on the use of CH‐EUS (46.3%), MRI (42.5%), or PET/TC (20.0%) as the preferred methods for follow‐up in patients who underwent EUS‐RFA.

In case of partial ablation or disease recurrence, 96.3% recommend retreatment with EUS‐RFA: of them, 15.0% suggested a maximum of a single EUS‐RFA retreatment, and 45.0% up to two sessions before changing treatment strategy.

### 3.5 Domain 5—Proposed Lexicon

The proposed EUS‐RFA lexicon, with related percentages of answers, is shown in Table 6. Similar proportions were found among highly experienced users as reported in Table S4.

**TABLE 6:** Proposed lexicon for endoscopic ultrasound‐guided radiofrequency ablation (EUS‐RFA).

	%
Definition of “Technical success”	
Achieving the presumed complete ablation of the neoplasm at the end of the procedure	88.8
The successful insertion of EUS‐RFA needle within the neoplasm	6.3
Other proposals	5.0
Definition of “Clinical success”	
Achieving symptoms control for at least 1 year in patients with insulinoma	92.3
Other proposals	7.7
Definition of “Complete ablation”	
Identification of the target lesion with no vascular enhancement	62.5
Complete disappearance of the target lesion	28.8
No uptake of gallium‐based tracers in case of pNENs	5.0
Other proposals	3.8
Definition of “Partial ablation”	
According to the RECIST criteria (at least a 30% decrease in the sum of diameters of target lesions)	38.0
At least 50% reduction of the longer axis measured on cross‐sectional imaging	35.4
Any reduction of the target lesion size	20.3
Other proposals	6.3
Definition of “Disease recurrence”	
Evidence of vascularized tumor tissue at any imaging examination (EUS, CT, MRI) following complete response	97.5
Evidence of neoplastic cells on EUS tissue sampling	2.5
Definition of “Post‐RFA pancreatitis”	
According to 2012‐revised Atlanta criteria for acute pancreatitis	48.8
According to ESGE criteria for post‐ERCP pancreatitis	38.8
According to CT‐scan criteria in patients with symptoms	12.5
Grading of severity of post‐RFA adverse events	
According to AGREE classification	45.0
According to ASGE lexicon	38.8
According to 2012‐revised Atlanta classification	15.0
Other proposals	1.3

Abbreviations: CT, computed tomography; ERCP, endoscopic retrograde cholangiopancreatography; EUS, endoscopic ultrasound; EUS‐RFA, endoscopic ultrasound‐guided radiofrequency ablation; MRI, Magnetic resonance imaging; pNENs, pancreatic neuroendocrine neoplasms.

## 4 Discussion

This international survey provides a comprehensive snapshot of current real‐world practice in EUS‐RFA for pancreatic neoplasms, highlighting substantial heterogeneity across virtually all domains of the procedure, even among experts. Despite increasing adoption of EUS‐RFA, the results clearly demonstrate a lack of standardized indications, procedural techniques, peri‐procedural management, follow‐up strategies, and shared terminology; moreover, they suggest significant heterogeneity across geographic regions. However, the present findings should be interpreted as preliminary and hypothesis‐generating, as they are derived from a single‐round survey without structured consensus methodology and are inherently subject to sampling and response biases.

Most physicians used EUS‐RFA in patients with insulinoma, reflecting the growing body of evidence supporting its safety and efficacy [2, 16, 17]. Prospective multicenter studies and comparative analyses have shown high rates of symptom control and favorable safety profiles, positioning EUS‐RFA as a valid alternative to surgical resection. Conversely, the proportion of answers declined for NF‐pNENs, underscoring persistent uncertainty regarding patient selection, tumor size thresholds, grading criteria, and oncologic adequacy. This lack of consensus mirrors the current absence of strong guideline endorsement and the limited availability of long‐term outcome data [4]. Significant regional differences support that EUS‐RFA practice is influenced by local factors. In particular, the higher use in PDAC in North America versus Europe is clinically relevant and likely reflects differences in guidelines, oncologic strategies, and institutional experience, with European recommendations generally discouraging ablation. These differences remained significant after adjustment, supporting their robustness; however, given the exploratory design, they should be considered hypothesis‐generating and require confirmation in larger, balanced cohorts [6, 7, 8]. For other indications than pNENs (i.e., PDAC, IPMNs, and metastases), our survey revealed either high variability, aligning with existing evidence, which remains confined to retrospective cohorts with heterogeneous study designs [19, 20, 29]. While recent studies suggest a potential palliative or cytoreductive role for EUS‐RFA in unresectable PDAC, the oncologic benefit remains uncertain, and safety concerns persist. As a result, oncology and gastroenterology guidelines do not suggest RFA in these settings. Indeed, the Italian Association of Medical Oncology suggests against local ablative techniques in patients with locally advanced PDAC [9]. In patients with pancreatic cystic neoplasms, the 2018 ACG guidelines do not recommend local ablation, whether by chemoablation or RFA, and suggest this option only for patients unfit for surgery or enrolled in clinical trials [10]; European guidelines and AIP Kyoto guidelines provide similar recommendations [11, 12].

There is a huge variability in EUS‐RFA technical aspects with no clear pattern on power settings, energy delivery duration, number of applications per session, probe length selection, and ablation strategy. This heterogeneity reflects the absence of standardized protocols and the biological diversity of pancreatic lesions. Available evidence suggests that tumor histology and tissue characteristics influence energy requirements: softer, well‐vascularized lesions, such as pNENs, respond to lower power settings, whereas fibrotic lesions, such as PDAC, often require higher energy [26, 30, 31, 32]. The lack of comparative studies precludes definitive recommendations, reinforcing the need for structured methodological standardization.

Peri‐procedural management also showed high variability and low standardization. No clear prevailing approach was observed on antibiotic prophylaxis for solid lesions and post‐RFA pancreatitis prophylaxis. Routine rectal NSAIDs administration reflects extrapolation from ERCP literature rather than RFA‐specific evidence [33]. While NSAIDs prevent post‐ERCP pancreatitis, their benefit is linked to ERCP‐related injury, driven by papillary manipulation, hydrostatic pressure, and contrast‐induced ductal irritation. In contrast, post‐EUS‐RFA pancreatitis is related to thermal injury with coagulative necrosis within the surrounding parenchyma. The prophylactic NSAIDs effect in EUS‐RFA should be demonstrated [34]. A striking discordance was observed regarding MPD proximity. While 97.5% of highly experienced users recognized the high‐risk nature of MPD involvement, reflecting findings from the RAFPAN study, no standardization of preventive strategies was observed [27]. This “risk‐mitigation gap” where risk is identified represents a critical vulnerability in current EUS‐RFA practice. Continued efforts should focus on comparing prophylactic stenting versus pharmacological prophylaxis in high‐risk cohorts.

Post‐procedural follow‐up and outcome assessment were also heterogeneous. There was no standardization of the imaging modality and timing of follow‐up, or definition of complete or partial ablation. Notably, while disease recurrence was clearly defined by most respondents as the reappearance of vascularized tissue on imaging, other key terms, including post‐RFA pancreatitis, lacked consensus. This semantic variability limits cross‐study comparability and impairs the interpretation of existing literature.

Interestingly, the similarity between highly experienced and non‐expert physicians suggests that variability in EUS‐RFA practice is not primarily driven by operator experience but rather reflects the absence of protocols and evidence‐based guidance. Future studies aiming to define EUS‐RFA competency thresholds are needed.

This study has several limitations. First, the geographic distribution of participants was inhomogeneous, with a predominance of Europeans; the practices and levels of standardization reported may not fully capture region‐specific approaches due to different clinical pathways and oncologic strategies. In addition, the limited number of respondents from North America and Asia results in small subgroups, reducing statistical power and affecting the reliability of comparisons; indeed, the limited representation of non‐European physicians may have influenced the reported indications. Moreover, despite internal evaluation rounds before sharing, the proposed questionnaire is not validated. Finally, the sampling strategy represents an additional limitation since a single manufacturer's registry list may have led to potential selection bias, further amplified by the supplementary recruitment strategy (“snowball effect” bias).

In conclusion, EUS‐RFA remains a rapidly evolving technique practiced without shared standards, even among experts. While innovation has outpaced evidence, current practice variability contributes to inconsistent outcomes and complicates study comparisons. These results support an international, multidisciplinary effort to develop standardized EUS‐RFA methodologies. In this context, the present study should be considered a preliminary step toward the development of standardized EUS‐RFA practice. By identifying key areas of variability, it provides a framework for future research priorities.

## Author Contributions

A.L., M.T., S.F.C., B.N., K.D.‐C.P.: conceptualization, project administration, writing – original draft Preparation; G.M., A.F., L.F.: data curation, formal analysis; P.F., S.L., T.K., N.T., S.G.K., R.F.H., P.G.A.: supervision, investigation, software; M.B., M.C.C.B., F.C., M.G., J.S., A.Y.B.T., T.O., B.K., V.D., P.V., S.L.: writing – review and editing.

## Funding

The authors have nothing to report.

## Disclosure

Artificial Intelligence (AI) generated content: No AI tool has been used at any moment of study design, conceptualization, analysis, and manuscript preparation.

## Ethics Statement

The authors have nothing to report.

## Consent

The authors have nothing to report.

## Conflicts of Interest

Andrea Lisotti received consultancy fees from Olympus, Boston Scientific, Taewoong, and Medi‐Globe; Pietro Fusaroli received consultancy fees from Olympus, Boston Scientific; Nirav Thosani received grant from NIH R01 Grant; Tawfik Khoury received support for attending meeting from Olympus; Paolo Giorgio Arcidiacono received honoraria from Boston, Pentax, and Fujifilm and is a member of the advisory board of Ambu and MediGlobe; Somashekar G. Krishna received consultancy from Mauna Kea Technologies and Taewoong Medical USA; Peter Vilmann received consultancy from MediGlobe GmBH; Bertrand Napoléon received honoraria from Cousin Endoscopy, French distributor for Taewoong devices; Khanh Do‐Cong Pham received consultancy from Cook, Fujifilm, Ovesco, Taewoong Medical and is a member of the advisory board of Olympus and Ambu. Matteo Tacelli, Stefano Francesco Crinò, Graziella Masciangelo, Marc Barthet, Maria Cristina Conti Bellocchi, Sarah Leblanc, Antonio Facciorusso, Fabrice Caillol, Marc Giovannini, Lorenzo Fuccio, Jayanta Samanta, Anthony Y.B. Teoh, Takeshi Ogura, Roald Flesland Havre; Bojan Kovacevic, Vinay Dhir, and Sundeep Lakhtakia declare no conflicts of interest.

## Supporting information


**Table S1:** Indication for EUS‐RFA according to region of origin.
**Table S2:** EUS‐RFA indications in patients suffering from pancreatic adenocarcinoma, metastases and intraductal papillary mucinous neoplasms.
**Table S3:** Management of endoscopic ultrasound‐guided radiofrequency ablation in case of pancreatic neoplasms located close the main pancreatic duct, with or without involvement or upstream dilation.
**Table S4:** Proposed lexicon for endoscopic ultrasound‐guided radiofrequency ablation (EUS‐RFA) by highly experienced operators*.

**Appendix S1:** Invitation email.
**Appendix S2:** Online survey text and questions.

**Data S1:** Supporting information.

## Data Availability

The data that support the findings of this study are available on request from the corresponding author. The data are not publicly available due to privacy or ethical restrictions.
