# Supplementary material for: Methodology Assessment of Endoscopic Ultrasound Radiofrequency Ablation (EUS‐RFA) for Pancreatic Neoplasms: Results From an International Survey
Source: Dig Endosc. 2026 Jun 29;38(7):e70212. doi: 10.1111/den.70212 (PMC13312040; doi:10.1111/den.70212)
Supplement: Supplementary file 1 — Table S1: Indication for EUS‐RFA according to region of origin. Table S2: EUS‐RFA indications in patients suffering from pancreatic adenocarcinoma, metastases and intraductal papillary mucinous neoplasms. Table S3: Management of endoscopic ultrasound‐guided radiofrequency ablation in case of pancreatic neoplasms located close the main pancreatic duct, with or without involvement or upstream dilation. Table S4: Proposed lexicon for endoscopic ultrasound‐guided radiofrequency ablation (EUS‐RFA) by highly experienced operators*. [file DEN-38-0-s003.docx]

**Supplementary Table 1** – Indication for EUS-RFA according to region of origin

| **Indication for EUS-RFA** | **Europe**  (n=67) | **America**  (n=12) | **Asia**  (n=11) | **Chi square**  **P Value** | **Bonferroni-adjusted**  **P value** |
| --- | --- | --- | --- | --- | --- |
| Insulinoma | 96.9% | 75.0% | 100% | 0.006 | **0.038** |
| Other functioning pNENs | 53.1% | 50.0% | 66.7% | 0.785 | 1.000 |
| Non functioning pNENs | 67.2% | 91.7% | 77.8% | 0.164 | 0.982 |
| Pancreatic metastases | 60.9% | 58.3% | 33.3% | 0.303 | 1.000 |
| PDAC | 26.6% | 83.3% | 55.6% | <0.001 | **0.003** |
| IPMN | 29.7% | 50.0% | 22.2% | 0.234 | 1.000 |
| Liver tumors | 17.2% | 33.3% | 44.4% | 0.092 | 0.64 |
| Abdominal lymph node | 15.6% | 8.3% | 0.0% | 0.340 | 1.000 |
| Other solid pancreatic neoplasms | 6.3% | 33.3% | 11.1% | 0.014 | 0.100 |
| Other pancreatic cystic neoplasms | 6.3% | 33.3% | 11.1% | 0.014 | 0.100 |
| Adrenal gland tumor | 12.5% | 0.0% | 11.1% | 0.440 | 1.000 |
| Solid pseudopapillary tumor | 9.4% | 16.7% | 0.0% | 0.370 | 1.000 |
| Celiac plexus RFA ablation | 18.8% | 41.7% | 22.2% | 0.220 | 1.000 |

Unadjusted P values were calculated using chi-square testing. Bonferroni-adjusted P values were used for interpretation of statistical significance. Adjusted P values <0.05 were considered statistically significant.

Abbreviations: EUS-RFA – endoscopic ultrasound-guided radiofrequency ablation; pNENs - pancreatic neuroendocrine neoplasms; PDAC – pancreatic ductal adenocarcinoma; IPMN – intraductal papillary mucinous neoplasm.

**Supplementary Table 2** – EUS-RFA indications in patients suffering from pancreatic adenocarcinoma, metastases and intraductal papillary mucinous neoplasms.

| **Pancreatic Metastases** | | **Pancreatic ductal adenocarcinoma** | | **Intraductal papillary mucinous neoplasms** | |
| --- | --- | --- | --- | --- | --- |
| *Indication for EUS.RFA* | *%* | *Indication for EUS.RFA* | *%* | *Indication for EUS.RFA* | *%* |
| Selected cases after MDT discussion | 71.4% | Selected cases after MDT discussion | 32.9% | Selected cases after MDT discussion | 35.3% |
| High-risk surgical patients | 39.3% | High-risk surgical patients with resectable neoplasms | 21.2% | High-risk surgical patients | 28.2% |
| Based on patients’ preference | 29.8% | Combined to chemotherapy with neoadjuvant intent | 12.9% | Based on patients’ preference | 12.9% |
| In case of disease limited to pancreatic parenchyma | 41.7% | After neoadjuvant therapy, if the neoplasm is still not resectable | 17.7% | Always, in case of enhanced mural nodule | 0% |
| Only within research protocols | 3.6% | In locally-advanced disease, with palliative intent | 24.7% | Only within research protocols | 1.2% |
| Never | 20.2% | In metastatic disease, with palliative intent | 17.7% | Never | 57.7% |
|  |  | Only within research protocols | 11.8% |  |  |
|  |  | Never | 50.6% |  |  |

Abbreviations: EUS-RFA – endoscopic ultrasound-guided radiofrequency ablation; MDT – multidisciplinary team; NEN – neuroendocrine neoplasm.

**Supplementary Table 3** – Management of endoscopic ultrasound-guided radiofrequency ablation in case of pancreatic neoplasms located close the main pancreatic duct, with or without involvement or upstream dilation.

| **Neoplasms close to the MPD**  **without involvement or upstream dilation** | | | **Neoplasms close to the MPD**  **with involvement or upstream dilation** | | |
| --- | --- | --- | --- | --- | --- |
| *Management* | *All users* | *Highly-experienced users** | *Management* | *All users* | *Highly-experienced users ** |
| Reducing duct damage (i.e., increase power setting, avoid duct puncture, or contact with the probe) | 42.5% | 40.5% | Reducing duct damage (i.e., increase power setting, avoid duct puncture, or contact with the probe) | 11.3% | 5.4% |
| Not changing the usual management | 13.8% | 21.6% | Not changing the usual management | 15.0% | 27.0% |
| Performing ERCP with prophylactic MPD stenting few days before EUS-RFA | 8.8% | 10.8% | Performing ERCP with prophylactic MPD stenting few days before EUS-RFA | 15.0% | 21.6% |
| Performing ERCP with prophylactic MPD stenting immediately before EUS-RFA | 15.0% | 13.5% | Performing ERCP with prophylactic MPD stenting immediately before EUS-RFA | 13.8% | 8.1% |
| Not performing EUS-RFA in these cases | 20.0% | 13.5% | Not performing EUS-RFA in these cases | 45.0% | 37.8% |

* ~~Expert~~ Highly-experienced users have been defined as operators with more than 20 EUS-RFA performed.

Abbreviations: MPD – main pancreatic duct; ERCP – endoscopic retrograde cholangiopancreatograpy; EUS-RFA - endoscopic ultrasound-guided radiofrequency ablation.

**Supplementary table 4** – Proposed lexicon for endoscopic ultrasound-guided radiofrequency ablation (EUS-RFA) by highly-experienced operators*

| **Definition of “Technical success”** | **(%)** |
| --- | --- |
| Achieving the presumed complete ablation of the neoplasm at the end of the procedure | 89.2% |
| The successful insertion of EUS-RFA needle within the neoplasm | 5.4% |
| Other proposals | 5.4% |
| **Definition of “Clinical Success”** | **(%)** |
| Achieving symptoms control for at least 1 year in patients with insulinoma | 91.7% |
| Other proposals | 8.3% |
| **Definition of “Complete ablation”** | **(%)** |
| Identification of the target lesion with no vascular enhancement | 51.3% |
| Complete disappearance of the target lesion | 40.5% |
| No uptake of gallium-based tracers in case of pNENs | 2.7% |
| Other proposals | 5.4% |
| **Definition of “Partial ablation”** | **(%)** |
| According to the RECIST criteria (at least a 30% decrease in the sum of diameters of target lesions) | 37.8% |
| At least 50% reduction of the longer axis measured on cross-sectional imaging | 43.2% |
| Any reduction of the target lesion size | 10.8% |
| Other proposals | 8.1% |
| **Definition of “Disease recurrence”** | **(%)** |
| Evidence of vascularized tumor tissue at any imaging examination (EUS, CT, MRI) following complete response | 97.3% |
| Evidence of neoplastic cells on EUS tissue sampling | 2.7% |
| **Definition of “Post-RFA pancreatitis”** | **(%)** |
| According to 2012-revised Atlanta criteria for acute pancreatitis | 43.2% |
| According to ESGE criteria for post-ERCP pancreatitis | 35.1% |
| According to CT-scan criteria in patients with symptoms | 21.6% |
| **Grading of severity of post-RFA adverse events** | **(%)** |
| According to AGREE classification | 43.2% |
| According to ASGE lexicon | 37.8% |
| According to 2012-revised Atlanta classification | 16.2% |
| Other proposals | 2.7% |

* ~~Expert~~ Highly-experienced users have been defined as operators with more than 20 EUS-RFA performed.

Abbreviations: EUS-RFA – endoscopic ultrasound-guided radiofrequency ablation; pNENs – pancreatic neuroendocrine neoplasms; CT – computed tomography; MRI – Magnetic resonance imaging; ERCP – endoscopic retrograde cholangiopancreatography; EUS – endoscopic ultrasound.
