# Supplementary material for: Methodology Assessment of Endoscopic Ultrasound Radiofrequency Ablation (EUS‐RFA) for Pancreatic Neoplasms: Results From an International Survey
Source: Dig Endosc. 2026 Jun 29;38(7):e70212. doi: 10.1111/den.70212 (PMC13312040; doi:10.1111/den.70212)
Supplement: Supplementary file 2 — Appendix S1: Invitation email. Appendix S2: Online survey text and questions. [file DEN-38-0-s001.docx]

Appendix 1 - Invitation email

Dear [Recipient’s Name],

I hope this email finds you well.

As part of an international effort to standardize and refine the methodology of endoscopic ultrasound radiofrequency ablation (EUS-RFA), we are inviting you to participate in a survey to gather expert opinions on current practices and technical aspects of this procedure.
EUS-RFA has recently gained increasing attention as a promising therapeutic approach, with growing evidence supporting its efficacy and safety. However, no standardized methodology has been established.  
Your expertise in the field would help shape a consensus on best practices. The survey should take approximately 15-20 minutes to complete and is accessible via the following link:

We would greatly appreciate your participation and any insights you can provide. If you have any questions or need further information, please feel free to contact me.

Responders will be invited to take part in a subsequent international Delphi consensus in the same field.

Thank you in advance for your time and contribution.

Best regards,  
Andrea Lisotti (Imola, Italy)

on behalf of the EUS-RFA Methodology study group

Stefano Francesco Crinò (Verona, Italy); Bertrand Napoléon (Lyon, France); Khanh Do-Cong Pham (Bergen, Norway)

**Appendix 2** – Online survey

**Implications for future research**

The results of this international survey highlight several critical gaps in the current evidence base for EUS-RFA and clearly delineate research priorities. First, beyond insulinoma, robust data on long-term clinical and oncological outcomes are lacking. Large, prospective, multicenter studies are urgently needed to evaluate the efficacy and durability of EUS-RFA in non-functioning pancreatic neuroendocrine neoplasms, pancreatic metastases, and selected pancreatic cystic neoplasms, with standardized inclusion criteria and uniform outcome definitions. Second, the wide heterogeneity observed in peri-procedural management underscores the need for dedicated studies to assess the effectiveness of prophylactic measures to reduce post-RFA adverse events, particularly acute pancreatitis and infections. Well-designed prospective registries or large retrospective cohorts could help clarify the role of antibiotic prophylaxis, rectal nonsteroidal anti-inflammatory drugs, aggressive hydration, and ductal protection strategies in specific clinical scenarios. Third, the lack of consensus on technical parameters—including power settings, energy delivery duration, probe length selection, and ablation strategy—underscores the need for comparative studies evaluating different ablative protocols. Such studies should ideally stratify patients by tumor histology, size, location, and proximity to critical structures to identify tailored, reproducible technical standards. Finally, the potential role of EUS-RFA as a complementary or palliative treatment in patients with pancreatic ductal adenocarcinoma remains exploratory. Future prospective studies, designed in close collaboration with oncologists and immunologists, are required to assess not only safety and local tumor control but also interactions with systemic therapies and potential immunomodulatory effects. Overall, the data generated by this survey provide a solid foundation for developing an international, multidisciplinary Delphi consensus to standardize indications, methodology, peri-procedural management, follow-up strategies, and terminology for EUS-RFA. Such standardization is essential for enabling meaningful comparisons across studies and for supporting the design of high-quality prospective trials.
