# Supplementary material for: Methodology Assessment of Endoscopic Ultrasound Radiofrequency Ablation (EUS‐RFA) for Pancreatic Neoplasms: Results From an International Survey
Source: Dig Endosc. 2026 Jun 29;38(7):e70212. doi: 10.1111/den.70212 (PMC13312040; doi:10.1111/den.70212)
Supplement: Supplementary file 3 — Data S1: Supporting information. [file DEN-38-0-s002.pdf]

EUS-RFA Methodology

Part I - Physician information

Detailed information on physician's background and experience

1. Name

2. Middle name (initials)

3. Surname

4. Affiliation

5. Email address

6. Medical specialization

- ◯ Gastroenterology and GI Endoscopy
- ◯ Interventional Radiology
- ◯ Surgery
- ◯ Oncology
- ◯ Other (please specify)

7. Endoscopic ultrasound-guided tissue sampling experience

- ◯ < 5 years
- ◯ 5 - 10 years
- ◯ > 10 years

8. EUS-guided tissue sampling/year

- ◯ < 50 EUS/year
- ◯ 50-100 EUS/year
- ◯ > 100 EUS/year

9. No. EUS radiofrequency ablation (EUS-RFA) performed (overall)

- ○ < 10 EUS-RFA procedures
- ○ 10 - 20 EUS-RFA procedures
- ○ 20 - 50 EUS-RFA procedures
- ○ > 50 EUS-RFA procedures

10. EUS-RFA volume/year

- ○ < 5 EUS-RFA
- ○ 5 - 10 EUS-RFA
- ○ > 10 EUS-RFA

EUS-RFA Methodology

Part II - Local facility

Detailed information on facility and local volume

11. Region

- ◯ Africa
- ◯ Asia
- ◯ Australia
- ◯ Europe
- ◯ Northern America
- ◯ Southern America

12. Institution

- ◯ University Hospital
- ◯ Public Hospital
- ◯ Private Hospital
- ◯ Other (please specify)

13. Country

14. At your facility, it is available (multiple answers allowed):

- ☐ 24/7 Radiology (CT scan)
- ☐ Interventional radiology
- ☐ Gastroenterology ward
- ☐ Gastrointestinal endoscopy
- ☐ ERCP
- ☐ 24/7 on-call GI endoscopy
- ☐ General surgery
- ☐ Emergency surgery
- ☐ Pancreatic surgery
- ☐ Oncology
- ☐ Pathology
- ☐ Endocrinology
- ☐ Nuclear medicine
- ☐ Multidisciplinary team (dedicated for GI neoplasms)
- ☐ Multidisciplinary team (dedicated for pancreatic neoplasms)
- ☐ Multidisciplinary team (dedicated for neuroendocrine neoplasms)

15. Is your Unit accreditated as a Center of Excellence for Neuroendocrine Tumor

- Yes
- No

16. If Yes, to which Society (i.e., ENETS, NANETS, other)

17. EUS volume/year (Institution)

- < 250 EUS/year
- 250 - 500 EUS/year
- 501 - 1000 EUS/year
- > 1000 EUS/year

18. No. EUS-RFA operators

- 1
- 2
- 3 or more

19. No. EUS-RFA/year (Institution)

- < 5 EUS-RFA per year
- 5 - 10 EUS-RFA per year
- 10 - 15 EUS-RFA per year
- > 15 EUS-RFA per year

EUS-RFA Methodology

Part III - Indications for EUS-RFA

Detailing which are the indications for EUS-RFA in your practice

20. Indications for EUS-RFA in your practice (multiple answers allowed):

- [ ] Insulinoma
- [ ] Other functioning pNENs (F-pNENs), such as gastrinoma, VIPoma, etc.
- [ ] Non-functioning pNENs (NF-pNENs)
- [ ] Pancreatic ductal adenocarcinoma (PDAC)
- [ ] Pancreatic metastases
- [ ] Solid pseudopapillary neoplasms (SPN)
- [ ] IPMN with mural nodules (MN)
- [ ] Other pancreatic solid neoplasms
- [ ] Other pancreatic cystic neoplasms
- [ ] Adrenal gland tumors
- [ ] Liver tumors, including metastases
- [ ] Other (please specify)

21. Do you perform any other EUS ablation for pancreatic neoplasms (i.e. EUS ethanol injection, microwave ablation, etc.)?

- ○ No
- ○ Yes, please mention the type of procedures

22. Do you perform EUS celiac plexus RFA?

- ○ Yes
- ○ No

23. When do you perform EUS-RFA in patients with functioning pNENs (multiple answers allowed):

- [ ] Always
- [ ] High-risk surgical patients
- [ ] Selected cases after multidisciplinary discussion
- [ ] Based on patient's preference
- [ ] Only within research protocols
- [ ] I do not treat F-NENs

24. When do you perform EUS-RFA in patients with non-functioning pNENs (multiple answers allowed):

- ☐ pNENs with an indication for surgery but high-risk surgical patients
- ☐ Selected cases after multidisciplinary discussion
- ☐ pNENs with increasing tumor size but still ≤20mm
- ☐ Based on patient's preference
- ☐ Only within research protocols
- ☐ I do not treat NF-NENs

25. When do you perform EUS-RFA in patients with non-functioning pNENs:

- ○ Only G1 pNENs
- ○ Both G1-G2 pNENs
- ○ I treat pNENs with EUS-RFA independently from grading

26. Is there a minimum size (mm) for performing EUS-RFA for non-functioning pNENs?

[ ]

27. Is there a maximum size (mm) for performing EUS-RFA for non-functioning pNENs?

[ ]

28. When do you perform EUS-RFA in patients with pancreatic adenocarcinoma (multiple answers allowed)?

- ☐ In high-risk surgical patients with resectable neoplasms
- ☐ Combined to chemotherapy with neoadjuvant intent
- ☐ After neoadjuvant chemo-radiotherapy if the tumor is still non-resectable
- ☐ In locally-advanced disease, with palliative intent (i.e., pain)
- ☐ In metastatic disease, with palliative intent (1.e., pain)
- ☐ Selected cases after multidisciplinary discussion
- ☐ Only within research protocols
- ☐ I do not treat patients with PDAC

29. When do you perform EUS-RFA in patients with pancreatic metastases from renal cell carcinoma (multiple answers allowed)?

- ☐ In case of disease limited to pancreatic parenchyma
- ☐ In case of high-risk surgical patients
- ☐ Based on patients' preference
- ☐ Selected cases after multidisciplinary discussion
- ☐ Only within research protocols
- ☐ I do not treat patients with pancreatic metastases

30. When do you perform EUS-RFA in patients with solid pseudopapillary neoplasm (multiple answers allowed)?

- [ ] Always, in case of small (<20 mm) neoplasm
- [ ] In case of high-risk surgical patients
- [ ] Selected cases after multidisciplinary discussion
- [ ] Based on patients' preference
- [ ] Only within research protocols
- [ ] I do not treat patients with SPN

31. When do you perform EUS-RFA in patients with IPMN with mural nodules (multiple answers allowed)?

- [ ] Always
- [ ] In case of high-risk surgical patients
- [ ] Selected cases after multidisciplinary discussion
- [ ] Based on patients' preference
- [ ] Only within research protocols
- [ ] I do not treat patients with IPMN with MN

32. Do you treat patients with other pancreatic cystic neoplasms?

- ( ) No
- ( ) Yes

Please specify

[ ]

EUS-RFA Methodology

Part IV - Procedural details

Clarifying the usual approach for EUS-RFA procedure in your practice

33. Which type of sedation is usually administered during EUS-RFA procedures?

- Moderate (conscious) sedation without anesthesiologist
- Deep sedation without anesthesiologist
- Deep sedation or general anesthesia upon anesthesiologist preference
- General anesthesia

34. Do you consider it recommended to perform EUS-RFA in an X-ray-equipped endoscopic room?

- Yes
- No

35. Do you perform EUS-RFA in an outpatient setting?

- Yes
- Only in selected cases
- No, only inpatient setting (due to reimbursement issues)
- No, only inpatient setting (due to clinical considerations)

36. Do you administer routinely antibiotic prophylaxis?

- Yes, always
- Only in patients with cystic neoplasms
- No

37. Do you administer prophylaxis for post-procedure pancreatitis?

- Yes, all patients receive both aggressive hydration and rectal NSAIDs
- Yes, all patient receive aggressive hydration
- Yes, all patient receive rectal NSAIDs
- No

38. When are you concerned about pancreatic duct injury?

- In case of lesions located ≤2 mm from the pancreatic duct
- In case of lesions located ≤1 mm from the pancreatic duct
- In case of lesions involving the pancreatic duct
- I am never concerned about post-RFA pancreatic duct injury

39. How do you manage EUS-RFA of pancreatic neoplasms located close to the Wirsung duct, without involvement or upstream dilation?

- ◯ I do not change the usual management
- ◯ I try to reduce duct damage (i.e., increase power setting, avoid duct puncture, or contact with the probe)
- ◯ I perform ERCP with pancreatic duct stenting few days before EUS-RFA
- ◯ I perform ERCP with pancreatic duct stenting immediately before EUS-RFA
- ◯ I do not perform EUS-RFA in these cases

40. How do you manage EUS-RFA of pancreatic neoplasms with Wirsung duct involvement and upstream dilation?

- ◯ I do not change the usual management
- ◯ I try to reduce duct damage (i.e., increase power setting, avoid duct puncture, or contact with the probe)
- ◯ I perform ERCP with pancreatic duct stenting few days before EUS-RFA
- ◯ I perform ERCP with pancreatic duct stenting immediately before EUS-RFA
- ◯ I do not perform EUS-RFA in these cases

41. Do you routinely perform contrast enhancement EUS (CH-EUS)?

- ◯ Yes, before and after EUS-RFA procedure
- ◯ Only before EUS-RFA, at the time of the diagnosis
- ◯ Only after EUS-RFA, to assess the presence of residual vital tissue
- ◯ No

42. Which ultrasound contrast agent do you use for CH-EUS (multiple answers allowed)?

- ☐ SonoVue®
- ☐ Sonazoid™
- ☐ Other (please specify)

____

43. How long do you wait before contrast administration after EUS-RFA?

- ◯ I do not perform CH-EUS after EUS-RFA
- ◯ I perform CH-EUS immediately after EUS-RFA
- ◯ I perform CH-EUS 1-3 minutes after EUS-RFA
- ◯ I perform CH-EUS 3-5 minutes after EUS-RFA
- ◯ I perform CH-EUS >5 minutes after EUS-RFA

44. Do you always use the same power setting for all EUS-RFA indications?

- ◯ No
- ◯ Yes (please specify the power in Watts)

____

45. Which power setting do you use for EUS-RFA in patients with pNENs?

46. Which power setting do you use for EUS-RFA in patients with cystic neoplasms?

47. Which power setting do you use for EUS-RFA in patients with adenocarcinoma?

48. Which power setting do you use for EUS-RFA in patients with other solid pancreatic neoplasms?

49. Do you always use the same probe size (tip length) for all EUS-RFA?

- No
- Yes

50. Which probe do you use for EUS-RFA (multiple answers allowed)?

- 5 mm length
- 7 mm length
- 10 mm length
- 15 mm length
- Other (please specify)

51. Which technical strategy do you prefer?

- Multiple high-power quick shots
- Single/few low-power longer shot(s)
- I have not a predefined technical strategy

52. Is there a maximum number of applications for each EUS-RFA session?

- No, until all tissue seems ablated
- Yes (please specify)

53. Is there a maximum time for each EUS-RFA application?

- No, until the generator automatically stops or impedance rapidly increases
- I manually stop the generator when hyperechoic white bubbles appear within the lesion
- Yes, I stop after (seconds)

EUS-RFA Methodology

Part V - Post-procedural management

Clarifying the usual approach for post-EUS-RFA management in your practice

54. Do you routinely test serum biochemistry the same day of the procedure?

- ○ Yes
- ○ No

55. If yes, which lab test do you request the same day of the procedure (multiple answers allowed)?

- ☐ Complete blood cell count
- ☐ Amylase/lypase
- ☐ Fasting serum glucose (in all patients)
- ☐ Fasting serum glucose (only in patients treated for insulinoma)
- ☐ C-reactive protein
- ☐ Procalcitonin
- ☐ Liver function tests (AST, ALT, GGT, bilirubin)
- ☐ Creatinine, blood urea nitrogen (BUN), electrolytes
- ☐ Other (please specify)

56. Do you routinely test serum biochemistry the day after the procedure?

- ○ Yes
- ○ No

57. If yes, which lab test do you request the day after EUS-RFA (multiple answers allowed)?

- ☐ Complete blood cell count
- ☐ Amylase/lypase
- ☐ Fasting serum glucose (in all patients)
- ☐ Fasting serum glucose (only in patients treated for insulinoma)
- ☐ C-reactive protein
- ☐ Procalcitonin
- ☐ Liver function tests (AST, ALT, GGT, bilirubin)
- ☐ Creatinine, blood urea nitrogen (BUN), electrolytes
- ☐ Other (please specify)

58. Do you routinely perform a CT-scan with contrast medium before patients' discharge or within 2 weeks?

- ◯ Yes, routinely before discharge
- ◯ Yes, within 2 weeks to assess residual vital tissue or adverse events
- ◯ Only in case of suspected adverse events
- ◯ No

59. Which diagnostic method do you use for patients' follow-up (multiple answers allowed)?

- ☐ Trans-abdominal ultrasound
- ☐ CT-scan with contrast medium
- ☐ EUS without contrast agents
- ☐ EUS + CH-EUS
- ☐ MRI with contrast medium
- ☐ PET/TC with Ga-based tracers for pNENs
- ☐ PET/TC with 18F-FDG

60. What's the ideal timing for the first oncological follow-up in patients treated for pNENs?

- ◯ 1 month
- ◯ 3 months
- ◯ 6 months

61. In case of partial ablation or recurrence, how many EUS-RFA sessions do you perform before changing treatment strategy?

- ◯ 0 (in case of persistence or recurrence no indication for another EUS-RFA session)
- ◯ 1
- ◯ 2
- ◯ No maximum limit

Part VI - Proposed definitions and lexicon

Collect your proposal for a shared lexicon for EUS-RFA

62. How do you define EUS-RFA technical success?

- ◯ The successful insertion of EUS-RFA needle within the neoplasm
- ◯ Achieving the presumed complete ablation of the neoplasm at the end of the procedure
- ◯ Other (please specify)

63. How do you define EUS-RFA clinical success in patients treated for insulinoma?

- ◯ Achieving symptoms control for at least 1 year in patients with insulinoma
- ◯ Other (please specify)

64. How do you define complete ablation?

- ◯ Complete disappearance of the target lesion on cross-sectional imaging (CT, MRI)
- ◯ Complete disappearance of the target lesion on EUS
- ◯ Identification of the target lesion with no vascular enhancement on cross-sectional imaging (CT, MRI)
- ◯ Identification of the target lesion with no vascular enhancement on contrast-enhanced EUS
- ◯ No uptake of gallium-based tracers in case of pNENs
- ◯ Other (please specify)

65. How do you define partial ablation?

- ◯ Any reduction of the target lesion size
- ◯ At least 50% reduction of the longer axis measured on cross-sectional imaging
- ◯ According to the RECIST criteria (At least a 30% decrease in the sum of diameters of target lesions, taking as reference the baseline sum diameters)
- ◯ Other (please specify)

66. What is the optimal timing to define the ablation results (complete ablation, partial ablation, no response) in patients with pNENs?

- At 3 months
- At 6 months
- At 12 months
- 6 months after the last EUS-RFA session (in case of multiple sessions)
- Other (please specify)

67. How do you define disease recurrence?

- Evidence of vascularized tumor tissue at any imaging examination (EUS, CT, MRI) following complete response at the first follow-up
- Other (please specify)

68. How do you define post-EUS-RFA pancreatitis?

- According to 2012-revised Atlanta criteria for acute pancreatitis
- According to ESGE criteria for post-ERCP pancreatitis
- According to CT-scan criteria in patients with symptoms
- Other (please specify)

69. How do you propose to grade the severity of post-EUS-RFA adverse events?

- According to ASGE lexicon
- According to AGREE classification
- According to 2012-revised Atlanta classification
- Other (please specify)

70. Do you have any final suggestion or comment on these fields?
